# Differential Extraction and Preliminary Identification of Polyphenols from *Ugni candollei* (White Murta) Berries

**DOI:** 10.3390/antiox13060623

**Published:** 2024-05-21

**Authors:** Natalia Fuentes-Jorquera, Roberto I. Canales, José R. Pérez-Correa, Jara Pérez-Jiménez, María Salomé Mariotti-Celis

**Affiliations:** 1 Chemical and Bioprocess Engineering Department, School of Engineering, Pontificia Universidad Católica de Chile, Vicuña Mackenna 4860, P.O. Box 306, Santiago 7820436, Chile; nafuentes4@uc.cl (N.F.-J.); rocanalesm@uc.cl (R.I.C.); 2 Department of Metabolism and Nutrition, Institute of Food Science, Technology and Nutrition (ICTAN-CSIC), José Antonio Novais 10, 28040 Madrid, Spain; jara.perez@ictan.csic.es; 3 CIBER de Diabetes y Enfermedades Metabólicas Asociadas (CIBERDEM), Instituto de Salud Carlos III, 28040 Madrid, Spain; 4 School of Nutrition and Dietetics, Faculty of Medicine, Universidad Finis Terrae, Santiago 7501015, Chile

**Keywords:** antioxidants, deep eutectic solvents, hot pressurized water extraction, LC-MS, medicinal properties, native plants, polyphenols

## Abstract

*Ugni candollei*, commonly known as white murta, is a native Chilean berry with a polyphenol composition that has been underexplored. This study aimed to establish a comprehensive profile of white murta polyphenols using ultra-performance liquid chromatography electrospray ionization Orbitrap mass spectrometry (UPLC-ESI-ORBITRAP MS). Additionally, it compared the efficacy of conventional extraction methods with emerging techniques such as deep eutectic solvent (DES) extraction and hot pressurized water extraction (HPWE). The analysis tentatively identified 107 phenolic compounds (84 of them reported for the first time for this cultivar), including 25 phenolic acids, 37 anthocyanins, and 45 flavonoids. Among the prominent and previously unreported polyphenols are ellagic acid acetyl-xyloside, 3-p-coumaroylquinic acid, cyanidin 3-*O*-(6′-caffeoyl-glucoside, and phloretin 2′-*O*-xylosyl-glucoside. The study found HPWE to be a promising alternative to traditional extraction of hydroxybenzoic acids, while DES extraction was less effective across all categories. The findings reveal that white murta possesses diverse phenolic compounds, potentially linked to various biological activities.

## 1. Introduction

Polyphenols, secondary metabolites prevalent in the plant kingdom, are essential components of plant cell walls. They play a pivotal role in plant defense, protecting against oxidative stress and ultraviolet radiation [1]. Additionally, they show several health-promoting bioactivities, such as the neutralization of free radicals [2] or anti-inflammatory activities [3], which eventually lead to a potential risk reduction of several diseases, such as cancer [4] or type 2 diabetes [5].

Polyphenols originate from phenylpropanoid and phenylpropanoid acetate main chains and are chemically classified according to the number of phenolic rings and the structural elements connecting them. Thus, they encompass several subclasses, including phenolic acids, flavonoids (flavonols, flavanols, isoflavones, anthocyanins, and flavanones), stilbenes, and lignans [6]. These phytochemicals pervade our diet and are found in free and conjugated (also called non-extractable) forms in cereals, legumes, herbs, vegetables, nuts, and fruits [7,8]. In particular, fruits, including berries, are abundant in flavonoids in the form of esters and glucosides.

Murta, Murtilla, or Uñi in the Mapuche language, a prominent member of the Myrtaceae family, is a wild native perennial plant in south-central Chile, of which three varieties may be mostly found (*Ugni molinae*, *Ugni candollei,* and *Ugni selkirkii*). Murta fruit and leaf infusions are widely utilized in traditional medicine for their anti-inflammatory and analgesic properties [9]. Additionally, murta fruit extracts applied to cancer cell lines (SW48 and HT-29) induced cytotoxicity and apoptosis in human colorectal cancer cells after 72 h [10].

Previous studies on the phenolic composition of murta leaves and fruits have mostly explored red murta, *Ugni molinae*, revealing a variety of phenolic compounds. Thus, more than 30 anthocyanins, which are characteristics of berries, were detected by HPLC-MS [11]. A long list of flavonols has also been reported for this fruit [12,13]. While other authors have identified polyphenols belonging to other classes, such as phenolic acid, flavanols, or ellagitannins [14]. Also, a high correlation was reported between the antioxidant capacity and the total polyphenol content of murta fruit extracts [15]. The effects of different drying methods on the phenolic profile of *Ugni molinae* were also compared [16]. Another study [17] focused on murta seeds (*Ugni molinae*), reporting a phenolic composition based on phenolic acids, anthocyanidins, and flavonoid glycosides. However, the knowledge of the bioactive constituents and associated biological activities of *Ugni molinae* is still limited [18]; therefore, more research is needed on this species.

Only a few studies have considered wild white murta (*Ugni candollei*). Thus, Avello et al. [19] measured the total phenolic and flavonoid content in the three varieties of murta (*Ugni molinae*, *Ugni candollei,* and *Ugni selkirkii*), finding the highest value for wild white murta. In another study, Avello et al. [20] characterized the phenolic profile of the three varieties by HPLC-ESI-MS analysis, being the only study providing a detailed phenolic composition of wild white murta. In particular, *white murta* was distinguished by its flavonoid content, including quercetin glycosides and their derivatives. This study also evaluated the antioxidant capacity of the extracts using the DPPH, ABTS, and hydroxyl radical stabilization methods, showing that *U. candollei* and *U. selkirkii* exhibited an antioxidant capacity higher than *U. molinae*. [19,20]. Since the polyphenol profile of a certain cultivar may vary due to location or climatic conditions, more studies on the phenolic profile of wild white murta are needed. Moreover, this approach should include more advanced extraction methods for a more complete investigation of the polyphenolic profile.

Hot pressurized liquid extraction (HPLE) is an efficient and green method for isolating biomolecules, especially polyphenols [21]. This method operates under high pressures (between 3.3 and 20.3 MPa) and temperatures (between 40 and 200 °C), conditions that enhance polyphenol solubility and reduce solvent viscosity, which accelerates dissolution and increases mass transfer rates [22]. These conditions also reduce solvent surface tension, enabling the solvent to penetrate the matrix more effectively, altering and disintegrating its structure. For instance, in hot pressurized water extraction (HPWE), water heated above 100 °C and pressurized beyond atmospheric conditions behaves like organic solvents due to a decreased dielectric constant.

Deep eutectic solvents (DES) have attracted interest as an alternative extraction procedure in recent years. DES are a mixture of a halide salt or other hydrogen bond acceptor (HBA) and a hydrogen bond donor (HBD). Chlorine chloride, the most commonly used HBA, can be combined with a suitable HBD (e.g., polyols, ethylene glycol, glycerol, and diols such as 1,2- and 1,3-propanediol) to produce mixtures that are liquid at room temperature and possess unique properties [23,24]. Compared to other solvents with similar physical properties, the main advantages of DES are low vapor pressure, flammability, and toxicity. At the same time, they are more stable and biodegradable. Moreover, they are easily prepared and customizable according to the specific application. It is important to consider that the efficiency of DES is pH dependent [25]. As with HPWE, DES have not been previously tested for extracting antioxidants from murta.

This study aims to characterize, using HPLC-ESI-ORBITRAP MS, the polyphenol profile of wild white murta (*Ugni candollei*) extracts, a native Chilean berry for which data on its phenolic profile obtained by advanced analytical techniques are still scarce. Hence, this study aims to increase our knowledge of the polyphenolic diversity of wild white murta and define the most suitable extraction strategies. Conventional extraction with organic solvents (acetone and methanol) was compared with green options such as deep eutectic solvents (prepared with ammonium salt and glycerol) and HPWE at different temperatures.

## 2. Materials and Methods

### 2.1. Chemicals

Acetone, methanol, water, glycerol, and choline chloride (all with 99% purity) were provided by Sigma-Aldrich Co. (St. Louis, MO, USA).

### 2.2. Solvents

Four solvents were used for the extractions: S1 (distilled water), S2 (acidified methanol/water, 50:50 *v*/*v*, pH = 2; 8 mL of 2N HCl volumetrically to 1 L with 50% *v*/*v* methanol/water), S3 (acetone/water, 70:30 *v*/*v*, pH = 7), and S4 (deep eutectic solvent, choline chloride:glycerol, 1:2 *w*/*w*, pH = 4).

### 2.3. Samples

Fruit samples of wild white murta (*Ugni candollei*) were collected in the forest of the Nahuelbuta Cordillera in April 2019 from Mahuilque (38°13′13.3′′ S 73°15′19.5′′ W), Biobío region of southern Chile. They were identified morphologically, considering taxonomic characteristics, such as leaf shape, fruit color, shoot color, and shape. The collected material was dry-cleaned, and the fruits were separated and frozen at −80 °C until freeze-drying (FDT-8620, Operon Co., Ltd., Gimpo, Republic of Korea). Finally, the dried samples were ground to 0.5 mm and stored at −20 °C until extraction.

### 2.4. Polyphenols Extraction

#### 2.4.1. Conventional Extraction

These extractions were carried out in duplicate with solvents S2 and S3. The samples (0.5 g of ground freeze-dried white murta fruits) were placed into 50 mL falcon tubes containing 20 mL of the corresponding solvent (S2 or S3). Extractions were performed in a rotary shaker for one h at 28 °C, and then the mixtures were centrifuged at 6000 rpm for 10 min. Subsequently, after the extractions with solvents S2 (Conv_1) and S3 (Conv_2), the supernatant from each tube was transferred to a 25 mL flask containing the corresponding solvent. A 4.5 mL aliquot of the corresponding extract was pipetted from each flask into a 15 mL falcon tube and concentrated to 1.5 mL using nitrogen. Lastly, all these treated samples were filtered with a 0.45 µm PTFE syringe filter [26] before injecting them (10 µL each) into the Ultra-Performance Liquid Chromatography-Electrospray Ionization-Orbitrap Mass Spectrometry (UPLC-ESI-ORBITRAP MS).

#### 2.4.2. DES Extraction

Approximately 0.5 g of freeze-dried and ground white murta was weighed into 50 mL falcon tubes in duplicate. Twenty milliliters of 1:2 choline chloride:glycerol (prepared in a 1:2 molar ratio at a temperature of 80 °C for 60 min and 300 rpm until stable DES formation) was added, and the extraction was carried out on a shaker rotary for 1 h at room temperature (28 °C). The extracts were centrifuged at 6000 rpm for 10 min. Supernatants were diluted 1:4 with HPLC water, passed through 0.45 μm PTFE syringe filters, and injected into Orbitrap (10 μL).

#### 2.4.3. Hot Pressurized Water Extraction (HPWE)

Five grams of ground freeze-dried white murta fruits mixed with approximately 100 g of neutral quartz sand was placed in a 100 mL extraction cell. The extractions were performed in duplicate in an extractor (Dionex ASE 150, Thermo Fisher Scientific, Waltham, MA, USA) at two temperatures: 120 °C (ASE_120) and 80 °C (ASE_80). The other extraction conditions were pure water (S1) as the solvent, one cycle of 10 min, 60% washing volume, and P = 1500 psi. Both extracts were filtered with a 0.45 µm PTFE syringe filter and injected (10 µL) into the UPLC-ESI-ORBITRAP MS system.

### 2.5. Polyphenol Analysis by UPLC with Orbitrap Detector

Polyphenol analysis was conducted using a Dionex Ultimate 3000 ultra-performance liquid chromatography (UPLC) system with a mass spectrometry (MS) Orbitrap Exactive plus detector (Thermo Fisher, Waltham, MA, USA). A reverse-phase Acquity UPLC BEH C18 column (1.7 μm × 2.1 × 100 mm) was used. Our procedures were based on the methods of Liu et al. [27]. Polyphenols were tentatively identified using the dedicated software Thermo Xcalibur^TM^ 2.2, which compares theoretical m/z values with experimental m/z values and accepts error values below five ppm. Exceptionally, error values up to 10 ppm were accepted when the sample compound was determined at least in one of the four extracts with an error below five ppm and the same retention time.

Once a tentative identification was performed, areas for the different polyphenols were collected, expressed as arbitrary intensity units. Then, polyphenols were classified into four main families: hydroxycinnamic acids, hydroxybenzoic acids, flavonoids, and anthocyanins, allowing the comparison of extraction efficiency for each polyphenol family among the different extraction systems.

In addition, the relative area of each polyphenol family in the different extraction systems was calculated. For this purpose, the sum of the areas of the four families was 100 percent. This information was used to evaluate the distribution of polyphenol families in each extraction system and to determine whether each system could selectively extract certain classes of polyphenols, even if they were not the most efficient in absolute terms.

Finally, for a better understanding of the major compounds in murta and to compare different extraction systems, the relative abundance of the six major polyphenols in each family was calculated. In this calculation, the sum of the area of all identified polyphenols for that family was considered 100 percent.

### 2.6. Statistical Analysis

Area values for each compound of the corresponding polyphenol family were expressed as the mean ± standard deviation. Data processing was conducted using SPSS Statistics 23.0, which used a one-way analysis of variance with Tukey’s post hoc test.

## 3. Results

### 3.1. Phenolic Compounds Profile by HPLC-ESI-ORBITRAP MS Analysis

The complete UPLC-MS analysis of white murta provided the tentative identification of one hundred and seven phenolic compounds in the different murta extracts; the whole list is provided as Supplementary Information (Table S1). It should be highlighted that 84 of these compounds had not been previously reported for this cultivar. Overall, they corresponded to 25 phenolic acids (hydroxybenzoic and hydroxycinnamic acids), 45 flavonoids, and 37 anthocyanins (which are a class of flavonoids but, due to their observed relevance in this fruit, are considered separately). It should be mentioned that white murta, despite its name, exhibits a pink color associated with its anthocyanin content. All these compounds were identified with an error value below five ppm applied to the molecular weight of the identified compounds after searching for probable compounds based on previous literature on phenolic compounds in berries and, particularly, in red murta [11,12,13,14] and a couple of previous studies in white murta [20]. It should be mentioned that this exploration of white murta was focused on extractable polyphenols since they are the most studied and bioaccessible; however, all fruits exhibit a significant fraction of non-extractable polyphenols [28], which should be explored in further studies.

**Figure 1:**
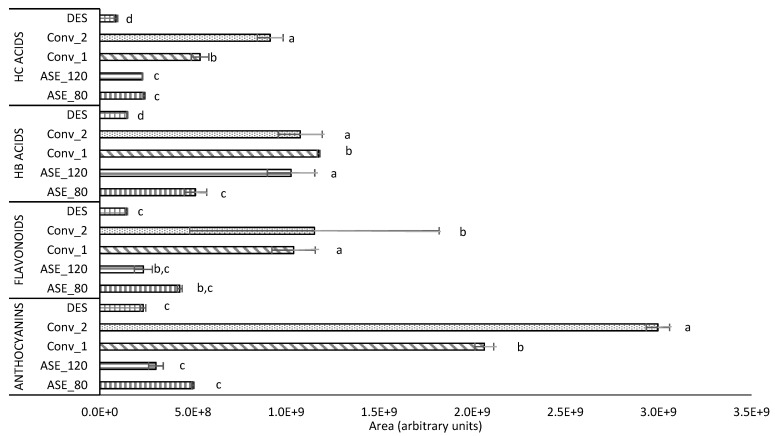
Sum of areas of the families of phenolic compounds tentatively identified after HPLC-MS analysis in white murta extracts obtained with different extraction procedures. Different letters (a, b, c, and d) represent statistical differences (*p* < 0.05) between extraction systems for the same polyphenol class. DES: deep eutectic solvent extract; ASE_120: extract with pressurized hot water at 120 °C; ASE_80: extract with pressurized hot water at 80 °C; Conv_1: extract with 50% aqueous methanol; Conv_2: extract with 70% aqueous acetone. HC, hydroxycinnamic acids; HB, hydroxybenzoic acids.

The overall comparison of the efficiency of the different extraction systems for each polyphenol family is depicted in Figure 1, while the distribution of the different families by each extraction system is shown in Table 1. Although the injection of standards would have allowed a quantification of the identified compounds, being this aspect a limitation of the study, the existing relationship between abundance and concentration allowed us to perform a general comparison of the efficiency of the different extraction approaches.

Overall, the two conventional extractions with organic solvents (Conv_1 and Conv_2) were the most efficient systems, providing the highest abundance of phenolic compounds, followed by the two HPWE extractions (ASE_80 and ASE_120), and finally by DES (Figure 1).

When the two conventional extraction systems were compared, no differences were observed for hydroxybenzoic acids and flavonoids, but Conv_2 proved to be the most effective in extracting anthocyanins and hydroxycinnamic acids (Figure 1). Regarding these results, the extraction efficiency of polyphenols depends on several factors, including the solvation characteristics of the solvent, the polyphenol structure, and the extraction temperature. Solvents’ solvation capacity is influenced by their polarity [29] and their ability to form hydrogen bonds with the metabolite of interest [30]. In this context, water-acetone and water–methanol mixtures are preferred for conventional polyphenol extraction due to their intermediate polarity and hydrogen bonding capabilities.

At the same time, it should not be disregarded that the food matrix strongly influences polyphenol extraction, combined with the mentioned factors. Thus, despite hydroxycinnamic acids having a carboxylic acid group contributing to their polarity, their overall structure may not be as hydrophilic as that of flavonoids. Consequently, there is a trade-off between the polarity of the compound and the properties of the food matrix, which explains, for example, why methanol is a better solvent for extracting chlorogenic acid from apples or why 50% acetone is more efficient than methanol alone or with water [31] for extracting the same compound from mulberry. The former is a fruit closer to white murta, and the result agrees with the ones reported here, with a higher efficiency of Conv_2 as compared to Conv_1. In the same way, although anthocyanins are commonly better extracted with methanol solutions than with acetone solutions, as found, for instance, for grape skin [32], the same study reported a higher extraction efficiency for acetone when the sample was grape pomace—the former result agrees with those reported in Figure 1 for anthocyanins in white murta.

Notably, the two HPWE systems exhibited distinct effects on the extraction efficiency of phenolic acids and flavonoids (including anthocyanins and other flavonoids). Thus, ASE_120 was the most efficient for hydroxybenzoic acids, while ASE_80 presented superior results for flavonoids and anthocyanins (and they behaved similarly regarding hydroxycinnamic acid extraction). In this sense, a similar tendency was reported for the recovery of gallic acid from grape pomace using subcritical water extraction, showing an approximately eightfold increase when the temperature was raised from 100 to 150 °C, likely attributed to the hydrolysis of galloylated compounds [33].

The flavonoid results contradict those previously reported for the HPWE extraction of Carménère pomace, where higher temperatures significantly increased their extraction. Previous research on various plant materials using water in subcritical conditions concluded that the optimum extraction temperature can vary depending on the flavonoid structure. Flavonoids with an OH side chain were optimally extracted at lower temperatures than those with O–CH3 and H side chains. Furthermore, the optimal temperatures for glycoside forms were lower than those for the less polar aglycones [34]. These aspects may have influenced the results obtained here. Finally, regarding anthocyanins, an optimization of HPWE carried out in raspberries showed, as found here, that an increase in temperature reduces their extraction yields [35]. Nevertheless, it should be highlighted that ASE_120, which is a green, food-grade, and efficient method, closely matched the performance of Conv_2 in extracting hydroxybenzoic acids from white murta, showing the potential of this technique, which might be improved with a specific optimization procedure.

As mentioned, the least efficient extraction system was DES, with the conditions tested here (choline chloride:glycerol, 1:2 *w*/*w*). These results agree with a recent study that compared the use of DES with conventional hydroalcoholic extraction for obtaining polyphenols from corn cobs [36]. Nevertheless, given the environmental advantages of using DES, further studies should explore whether extraction efficiency may be enhanced by substituting the hydrogen bond donor (HBD), such as ethylene glycol. A recent study with grape pomace showed that ethylene glycol surpasses glycerol in the recovery of phenolic acids and flavonols [37].

The results shown in Table 1 allow the comparison of the different extraction systems regarding quantitative efficiency for the different polyphenol classes. However, independent of the extraction efficiency, it is also relevant to assess whether some extraction systems are particularly useful for obtaining extracts enriched in a particular polyphenol class, i.e., it promotes a differential polyphenol extraction for further evaluation of biological activities. Thus, Table 1 compares the relative composition of the extracts obtained by each system. ASE_120 emerged as a good option for obtaining extracts enriched in hydroxybenzoic acids. Indeed, a study on a selection of medicinal and aromatic plants found that, in four out of the six analyzed samples, HPWE was more efficient for hydroxybenzoic acid extraction than conventional extraction [38]. Finally, Table 1 provides useful information about the repeatability of the results since it contains the values obtained by the two independent extractions performed with each system. It should be highlighted that, although DES was the less efficient system, at the same time, it was the one that showed the highest repeatability since higher variations were found for some polyphenol classes between the two extractions in both HPWE and conventional extraction. Other studies have also reported high repeatability of DES extraction, with a relative standard deviation of 3% and below, in a method developed for extracting curcumin from tea, honey, and spices [39]. In the case of HPWE, ASE_120 exhibited higher variability than ASE_80. These aspects should be considered if these approaches are used, for instance, for obtaining commercial extracts from white murta. 

### 3.2. Main Individual Phenolic Compounds per Family

**Figure 2:**
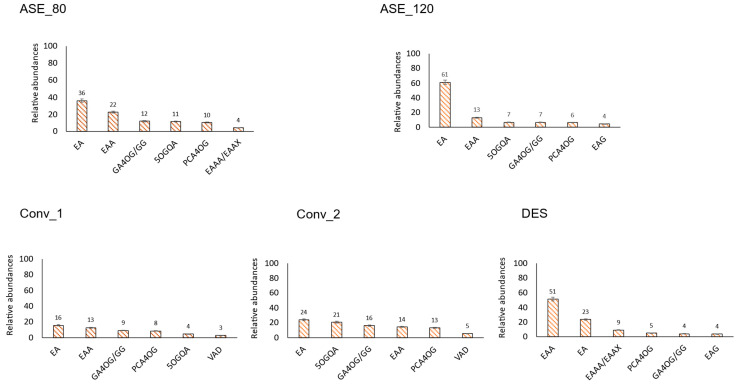
“White murta hydroxybenzoic acids”. Relative abundance, within the hydroxybenzoic acid family, of the six most abundant phenolic compounds tentatively identified in white murta extracts obtained with different extraction procedures. EAA: ellagic acid arabinoside, EA: ellagic acid, EAAA: ellagic acid acetyl-arabinoside, EAAX: ellagic acid acetyl-xyloside, PCA4OG: protocatechuic acid 4-O-glucoside, GA4OG: gallic acid 4-O-glucoside, GG: galloyl glucose, EAG: ellagic acid glucoside, 5OGQA: 5-O-galloylquinic acid, VAD: valoneic acid dilactone. DES: deep eutectic solvent extract; ASE_120: extract with pressurized hot water at 120 °C; ASE_80: extract with pressurized hot water at 80 °C; Conv_1: extract with 50% aqueous methanol; Conv_2: extract with 70% aqueous acetone.

**Figure 3:**
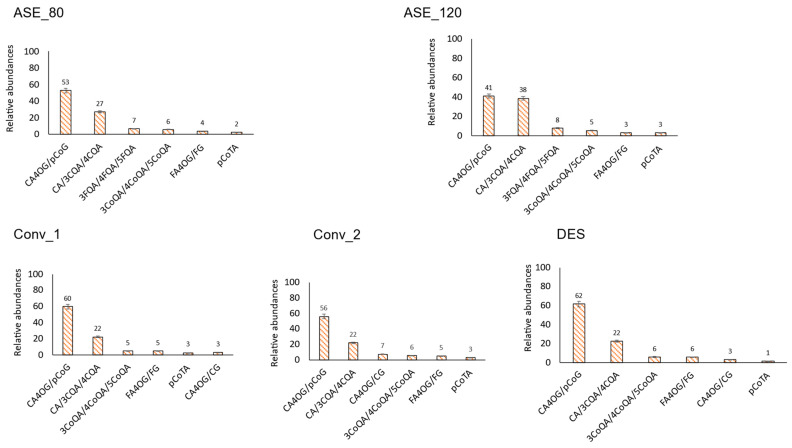
“White murta hydroxycinnamic acids”. Relative abundance, within the hydroxycinnamic acid family, of the six most abundant phenolic compounds tentatively identified in white murta extracts obtained with different extraction procedures. CA4OG: p-coumaric acid 4-O-glucoside, pCoG: p-coumaroyl glucose, CA: chlorogenic acid, 3CQA: 3-caffeoylquinic acid, 4CQA: 4-caffeoylquinic acid, 3CoQA: 3-p-coumaroylquinic acid, 4CoQA: 4-p-coumaroylquinic acid, 5CoQA: 5-p-coumaroylquinic acid, FA4OG: ferulic acid 4-O-glucoside, FG: feruloyl glucose, CA4OG: caffeic acid 4-O-glucoside, CG: caffeoyl glucose, pCoTA: p-coumaroyl tartaric acid, 3FQA: 3-feruloylquinic acid, 4FQA: 4-feruloylquinic acid, 5FQA: 5-feruloylquinic acid. DES: deep eutectic solvent extract; ASE_120: extract with pressurized hot water at 120 °C; ASE_80: extract with pressurized hot water at 80 °C; Conv_1: extract with 50% aqueous methanol; Conv_2: extract with 70% aqueous acetone.

**Figure 4:**
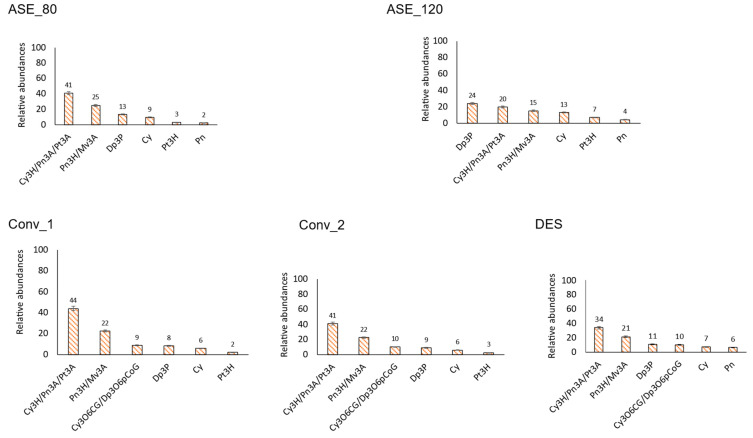
“White murta anthocyanins”. Relative abundance, within the anthocyanin family, of the six most abundant phenolic compounds tentatively identified in white murta extracts obtained with different extraction procedures. Cy3H: cyanidin 3-O-hexoside, Pn3A: peonidin 3-O-arabinoside, Pt3A: petunidin 3-O-arabinoside, Pn3H: peonidin 3-O-hexoside, Mv3A: malvidin 3-O-arabinoside, Dp3P: delphinidin 3-O-pentoside, Cy3O6CG: cyanidin 3-O-(6′-caffeoyl-glucoside), Dp3O6pCoG: delphinidin 3-O-(6′-p-coumaroyl-glucoside), Cy: cyanidin, Pn: peonidin, Pt3H: petunidin 3-O-hexoside. DES: deep eutectic solvent extract; ASE_120: extract with pressurized hot water at 120 °C; ASE_80: extract with pressurized hot water at 80 °C; Conv_1: extract with 50% aqueous methanol; Conv_2: extract with 70% aqueous acetone.

**Figure 5:**
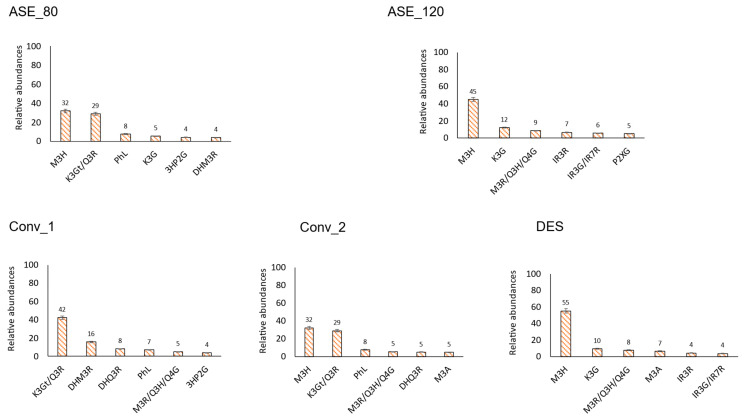
“White murta flavonoids”. Relative abundance, within the flavonoids family, of the six most abundant phenolic compounds tentatively identified in white murta extracts obtained with different extraction procedures. M3H: myricetin 3-O-hexoside, K3G: kaempferol 3-O-glucuronide, M3R: myricetin 3-O-rhamnoside, Q3H: quercetin 3-O-hexoside, Q4G: quercetin 4′-O-glucoside, M3A: myricetin 3-O-arabinoside, IR3R: isorhamnetin 3-O-rutinoside, IR3G: isorhamnetin 3-O-glucoside, IR7R: isorhamnetin 7-O-rhamnoside, P2XG: phloretin 2′-O-xylosyl-glucoside, K3Gt: kaempferol 3-O-galactoside, Q3R: quercetin 3-O-rhamnoside, 3HP2G: 3-hydroxyphloretin 2′-O-glucoside, DHM3R: dihydromyricetin 3-O-rhamnoside, DHQ3R: dihydroquercetin 3-O-rhamnoside, PhL: phloridzin. DES: deep eutectic solvent extract; ASE_120: extract with pressurized hot water at 120 °C; ASE_80: extract with pressurized hot water at 80 °C; Conv_1: extract with 50% aqueous methanol; Conv_2: extract with 70% aqueous acetone.

Due to the high number of phenolic compounds that were tentatively identified in this analysis, it was considered that a good strategy for better understanding the major compounds in murta, as well as comparing the different extraction systems, was to focus on the six major phenolic compounds in each family and extraction system, as depicted in Figure 2, Figure 3, Figure 4 and Figure 5; the full list of identified compounds is available as Supplementary Material (Table S1). These data are compared with those available in the literature.

#### 3.2.1. Hydroxybenzoic Acids

The hydroxybenzoic acids identified in white murta comprised gallic acid and derivatives, ellagic acid and derivatives, protocatechuic acid, punicalagin, and valoneic acid dilactone (Table S1). As shown in Figure 2, the search of the six most abundant compounds in each extraction procedure yielded four compounds that were present in all of them (ellagic acid, ellagic acid arabinoside, gallic acid 4-*O*-glucoside/ galloyl glucose, and protocatechuic acid 4-*O*-glucoside), as well as four compounds present only in some of the extracts: 5-*O*-galloylquinic acid (found in DES only as a minor compound), ellagic acid acetyl arabinoside-xyloside (found in ASE_80 as minor compound), ellagic acid glucoside (found in ASE 120 as a minor compound), and valoneic acid dilactone (found in DES, Conv_1 and Conv_2 as minor compounds). Avello et al. [20] reported galloylquinic acid, pentoside ellagic acid, and rhamnoside ellagic acid, confirming the presence of ellagic and quinic acid derivatives in the white murta profile.

As shown in Figure 1, in Conv_1, the election of the six most abundant hydroxybenzoic acids corresponded to about 50% of the detected signals for this class. At the same time, it was about 90% for the other extractions. In the cases of ASE_120 and DES, the proportion of the most concentrated hydroxybenzoic acid was much higher than the others. In contrast, in the other extraction techniques, the proportion of the six most abundant compounds was more similar, showing the potential of ASE_120 for the selective extraction of ferulic acid from white murta. Interestingly, this effect was achieved by applying a temperature for ASE that was not in the highest values reported for polyphenol extraction since the most common range is between 120 and 170 °C [40]. Thus, in this study, polyphenol extraction was achieved without using temperatures that can lead to the formation of undesirable compounds, such as Maillard reaction products [40]. In addition, the ionization properties of subcritical water change as the temperature rises, which can result in hydrolysis reactions of the natural components of the matrix. This limits the range of applicability of this technique [41].

It should be highlighted that punicalagin, an ellagitannin, was detected as a minor signal in three extracts (ASE_120, Conv_1, and Conv_2). This compound, although quite characteristic of pomegranate (belonging to the Myrtales order, such as white murta), has been found in other species, e.g., *Cistus parviflorus* [42]. Moreover, the presence of ellagic acid and its derivatives as major constituents in the hydroxybenzoic acid family in white murta, together with the fact that ellagitannins are known to be present in many berries, suggest the possible presence of other ellagitannins in white murta. Avello et al. [20] previously detected ellagitannin in their study of white murta. However, due to the high molecular weight of these compounds and their specific polarity, the conditions used here were not suitable for their extraction. Therefore, specific methods for ellagitannin extraction, as described by García-Villalba et al. [43], should be considered for future white murta analyses.

Some authors have reported the presence of hydroxybenzoic acids in red murta, such as gallic acid [44,45], ellagic and gallic acid derivatives [14], and protocatechuic acid [46], thus exhibiting a similar profile to white murta.

#### 3.2.2. Hydroxycinnamic Acids

The hydroxycinnamic acids identified in the white murta extracts correspond to derivatives of p-coumaric acids, ferulic acid, caffeic acid, and sinapic acid, including the associations with quinic acid (Table S1). Different isomers were suggested in this polyphenol class since it was impossible to assign an identity to them in this exhaustive exploratory analysis. Figure 3 shows the six most abundant signals for all the extractions. In all cases, p-coumaric acid, 4-*O*-glucoside/ p-coumaroyl glucose, and chlorogenic acid/ 3-caffeoylquinic acid/ 4-caffeoylquinic acid were the most abundant compounds in all extraction systems. Indeed, the proportion of total hydroxycinnamic acids corresponding to these two constituents was quite similar for all of them, ranging from 78% in Conv_2 to 84% in DES. Regarding the other most abundant hydroxycinnamic acids, three of them were also common to all extractions, although with differences in the ranking position (3-p-coumaroylquinic acid/ 4-p-coumaroylquinic acid/ 5-p-coumaroylquinic acid, ferulic acid 4-*O*-glucoside/ feruloyl glucose, and p-coumaroyl tartaric acid). In contrast, 3-feruloylquinic acid/ 4-feruloylquinic acid/ 5-feruloylquinic acid was most efficiently extracted with both HPWE extractions, while conventional and DES extractions were the most suitable ones for caffeic acid 4-*O*-glucoside/ caffeoyl glucose.

Caffeoylquinic acid and derivatives have been previously identified on white murta [20], while derivatives of ferulic acid, caffeic acid, and sinapic acid have been found in red murta [14]. Furthermore, Gómez-Pérez et al. [46] conducted a study on red murta, analyzing polyphenols in free and bound fractions. Subsequent HPLC analysis of specific compounds revealed the presence of p-coumaric acid in the bound fraction, which suggests that this phenolic acid, initially present in various derivatives, is likely released following the alkaline treatment of the sample.

#### 3.2.3. Anthocyanins

The thirty-nine identified anthocyanins correspond to derivatives (mostly hexosides or pentosides, with or without the association of a phenolic acid) of cyanidin, delphinidin, peonidin, petunidin, pelargonidin, and malvidin (Table S1). In this polyphenol class, variations in molecular weight exist between the different aglycones and the potential pentosides/hexosides associated with them. As a result, numerous compounds within this class may have different possible identities (Table S1).

Based on the extraction technique, the six most abundant anthocyanins and their distribution are shown in Figure 4. Four anthocyanins were present in all the extracts: Cyanidin 3-*O*-hexoside/ peonidin 3-*O*-arabinoside/ petunidin 3-*O*-arabinoside, peonidin 3-*O*-hexoside/ malvidin 3-*O*-arabinoside (these two compounds were the two most abundant ones in conventional and DES extractions), delphinidin 3-*O*-pentoside, and cyanidin. Additionally, petunidin 3-*O*-hexoside was present in conventional and HPWE extractions but not in DES; peonidin was detected in DES and HPWE extractions but not in conventional extractions; and delphinidin 3-*O*-(6′-p-coumaroyl-glucoside) was found in DES and conventional extractions but not in HPWE extractions. It should be mentioned that the six most abundant anthocyanins in white murta represented between 83% of all anthocyanins (ASE_120 extraction) and 91% (conventional extractions); thus, the other thirty-three detected anthocyanins were present as minor compounds. However, it can’t be ruled out that those minor compounds possess relevant bioactivity, like resveratrol in red wine.

In the case of red murta, a wide diversity of anthocyanins has also been reported [11,47]. Notably, they included derivatives from cyanidin, malvidin, delphinidin, peonidin, and petunidin but not from pelargonidin. However, several pelargonidin derivatives have been found in strawberries [48], making their presence in white murta plausible, although they were not found by Avello et al. [20]. One of these compounds, pelargonidin 3-*O*-glucoside, has been reported to be the strawberry anthocyanin with the highest anti-inflammatory activity [49]. Here, we detected a pelargonidin hexoside whose exact identity should be confirmed with MS/MS analysis or commercial standards.

#### 3.2.4. Other Flavonoids

The other flavonoids detected in white murta comprised mostly flavonols (kaempferol, quercetin, galangin, isorhamnetin, jaceidin, patuletin, spinacetin, 3-methoxysinensetin, and myricetin, as aglycones or as several derivatives), as well as small amounts of methoxyflavonols, dihydroflavonols (dihydroquercetin, dihydromyricetin, and derivatives), dihydrochalcones (phloretin and derivatives), and chalcones (butein) (Table S1).

In this class of polyphenols, the six most abundant compounds showed the greatest differences in each extraction system (Figure 5). Only one compound (myricetin 3-*O*-hexoside) was present in all extraction systems, probably because of its high concentration. It was the most abundant in all extraction systems, except in Conv_1, where it was the second most abundant. The other nine compounds were distributed among the different extraction systems without a clear tendency; they corresponded to kaempferol derivatives (kaempferol 3-*O*-glucuronide), myricetin derivatives (myricetin 3-*O*-arabinoside), isorhamnetin derivatives (isorhamnetin 3-*O*-rutinoside, isorhamnetin 3-*O*-glucoside/ isorhamnetin 7-*O*-rhamnoside), phloretin derivatives (phloridzin, 3-hydroxyphloretin 2′-*O*-glucoside), dihydromyricetin derivatives (dihydromyricetin 3-*O*-rhamnoside), dihydroquercetin derivatives (dihydroquercetin 3-*O*-rhamnoside) or other compounds where a single entity could not be assigned (myricetin 3-*O*-rhamnoside/ quercetin 3-*O*-hexoside/ quercetin 4′-*O*-glucoside, kaempferol 3-*O*-galactoside/ quercetin 3-*O*-rhamnoside). These compounds accounted for at least 80% of the polyphenol family in all extraction systems, implying a minor proportion of the more than 40 additional compounds detected (Table S1). Notably, in ASE_80 and Conv_2, the two major compounds corresponded to 60% of the fraction. The first compound contributed 42–55% of the content to the family in the other three extraction systems. It should be highlighted that in DES extraction, myricetin 3-*O*-hexoside corresponded to 55% of the fraction, showing the potential of this green technique for achieving extracts specifically enriched in this flavonol. However, the specific identity of the compound should be first ascertained, i.e., myricetin 3-*O*-glucoside or myricetin 3-*O*-galactoside; both flavonols have exhibited anti-inflammatory activities [50,51].

When comparing these results with other flavonoids detected in red murta, a variety of myricetin, quercetin, and kaempferol derivatives have been reported by several authors [14,52]. In white murta, some derivatives of quercetin and myricetin, mainly pentosides and hexosides, and also triterpenoids such as madecasic acid, asiatic acid, oleanoic acid glucoside, and oleanoloic acid glucoside [20] were found previously. Also, one study found phloridzin in an extract of red murta performed with an acidified methanol/water solution. Other flavonoids detected here have not been reported for red murta. Isorhamnetin derivatives have been found in wild and cultivated berry species [53].

## 4. Conclusions

This study represents a comprehensive analysis of the phenolic composition of white murta (*Ugni candollei*), a native Chilean berry. Using UPLC-MS coupled to an Orbitrap detector, a tentative list of 107 phenolic compounds in extracts obtained by various methods, including conventional extraction, deep eutectic solvent (DES) extraction, and hot pressurized water extraction (HPWE) was identified. Of these, 84 compounds are reported for the first time for *U. candollei*. These compounds consist primarily of hydroxybenzoic acids, hydroxycinnamic acids, anthocyanins (which contribute to the pink color of the fruit), and other flavonoids. Many of these compounds are also present in the closely related red murta (*Ugni molinae*); a few have been previously detected in white murta. This study highlights HPWE as an effective alternative to conventional methods for the extraction of hydroxybenzoic acids. DES extraction was generally less efficient, suggesting further research, particularly in optimizing the hydrogen bond donor (HBD) components to improve selectivity. Overall, this research highlights the complexity of the phenolic compound profile in white murta. Consequently, further research is required to establish its biological properties and bioavailability.

## Figures and Tables

**Table 1:** Relative abundances of the different polyphenol families in the white murta extracts. Mean values from two replicates ± standard deviation.

Class/Family	ASE_80	ASE_120	Conv_1	Conv_2	DES
Hydroxycinnamic acids	14.1 ± 3.1	12.8 ± 2.0	10.0 ± 3.4	16.1 ± 2.5	14.20 ± 0.1
Hydroxybenzoic acids	30.6 ± 1.2	57.3 ± 0.9	12.4 ± 3.1	18.9 ± 0.1	24.1 ± 1.1
Flavonoids	25.6 ± 9.7	13.1 ± 2.1	30.2 ± 5.1	12.3 ± 9.8	23.6 ± 0.3
Anthocyanins	29.8 ± 5.4	16.8 ± 0.8	47.4 ± 1.4	52.7 ± 7.1	38.1 ± 0.4

ASE_80, extract with HPWE at 80 °C; ASE_120, extract with HPWE at 120 °C, both types of extracts with S1; Conv_1, extract with S3; Conv_2, extract with S2; DES, extract with S4. The sum of each column equals 100.

## Data Availability

Original data are available upon request to the authors.

## Supplementary Materials

The following supporting information can be downloaded at: https://www.mdpi.com/article/10.3390/antiox13060623/s1, Table S1: Tentative identification of one hundred and seven phenolic compounds in the different murta extracts.
