# Supplementary material for: Differential Extraction and Preliminary Identification of Polyphenols from Ugni candollei (White Murta) Berries
Source: Antioxidants (Basel). 2024 May 21;13(6):623. doi: 10.3390/antiox13060623 (PMC11200422; doi:10.3390/antiox13060623)
Supplement: Supplementary file 1 [file antioxidants-13-00623-s001.zip › antioxidants-2988618-supplementary.pdf]

Supplementary Table S1: tentative identification of one hundred and seven phenolic c

ID	Tentative identification	m/z calc	Molecular formula
Hydroxybenzoic acids			
1	Ellagic acid	300,9990	$C_{14}H_6O_8$
2	Ellagic acid acetyl-arabinoside/Ellagic acid acetyl-xyloside	475,0518	$C_{21}H_{16}O_{13}$
3	Ellagic acid arabinoside	433,0413	$C_{19}H_{14}O_{12}$
4	Ellagic acid glucoside	463,0518	$C_{20}H_{16}O_{13}$
5	Gallic acid 3-O-gallate	321,0252	$C_{14}H_{10}O_9$
6	Gallic acid 4-O-glucoside/Galloyl glucose	331,0671	$C_{13}H_{16}O_{10}$
7	Protocatechuic acid 4-O-glucoside	315,0722	$C_{13}H_{16}O_9$
8	Punicalagin	1083,0593	$C_{48}H_{28}O_{30}$
9	Valoneic acid dilactone	469,0049	$C_{21}H_{10}O_{13}$
10	4-Hydroxybenzoic acid 4-O-glucoside	299,0772	$C_{13}H_{16}O_8$
11	5-O-Galloylquinic acid	343,0671	$C_{14}H_{16}O_{10}$
Hydroxycinnamic acids			
12	Caffeic acid 4-O-glucoside/Caffeoyl glucose	341,0878	$C_{15}H_{18}O_9$
13	Chlorogenic acid/3-Caffeoylquinic acid/4-Caffeoylquinic acid	353,0878	$C_{16}H_{18}O_9$
14	Ferulic acid 4-O-glucoside/Feruloyl glucose	355,1035	$C_{16}H_{20}O_9$
15	p-Coumaroyl tartaric acid	295,0459	$C_{13}H_{12}O_8$
16	p-Coumaric acid 4-O-glucoside/p-Coumaroyl glucose	325,0929	$C_{15}H_{18}O_8$
17	3-p-Coumaroylquinic acid/4-p-Coumaroylquinic acid/5-p-Coumaroylquinic acid	337,0929	$C_{16}H_{18}O_8$
18	3-Feruloylquinic acid/4-Feruloylquinic acid/5-Feruloylquinic acid	367,1035	$C_{17}H_{20}O_9$

19	3-Sinapoylquinic acid/4-Sinapoylquinic acid/5-Sinapoylquinic acid	397,1140	$C_{18}H_{22}O_{10}$
20	3,4-Dicaffeoylquinic acid/3,5-Dicaffeoylquinic acid/4,5-Dicaffeoylquinic acid	515,1195	$C_{25}H_{24}O_{12}$
21	1,2-Diferuloylgentiobiose	693,2036	$C_{32}H_{38}O_{17}$
22	1,2-Disinapoylgentiobiose	753,2248	$C_{34}H_{42}O1_{9}$
23	1-Sinapoyl-2,2'-diferuloylgentiobiose	899,2615	$C_{43}H_{48}O_{21}$
24	1,2'-Disinapoyl-2-feruloylgentiobiose	929,2721	$C_{44}H_{50}O_{22}$
25	1,2,2'-Trisinapoylgentiobiose	959,2827	$C_{45}H_{52}O_{23}$
Flavonoids			
26	Butein	271,0612	$C_{15}H_{12}O_{5}$
27	Dihydroquercetin	303,0510	$C_{15}H_{12}O_{7}$
28	Dihydroquercetin 3-O-rhamnoside	449,1089	$C_{21}H_{22}O_{11}$
29	Dihydromyricetin 3-O-rhamnoside	465,1039	$C_{21}H_{22}O_{12}$
30	Galangin	269,0456	$C_{15}H_{10}O_{5}$
31	Isorhamnetin/Rhamnetin	315,0510	$C_{16}H_{12}O_{7}$
32	Isorhamnetin 3-O-rutinoside	461,1089	$C_{22}H_{22}O_{11}$
33	Isorhamnetin 3-O-glucoside/Isorhamnetin 7-O-rhamnoside	477,1039	$C_{22}H_{22}O_{12}$
34	Isorhamnetin-3-O-glucoside-7-O-rhamnoside	623,1618	$C_{28}H_{32}O_{16}$
35	Jaceidin 4'-O-glucuronide	535,1093	$C_{24}H_{24}O_{14}$
36	Kaempferol	285,0405	$C_{15}H_{10}O_{6}$
37	Kaempferol 3-O-rhamnoside	430,0905	$C_{21}H_{19}O_{10}$
38	Kaempferol 7-O-glucoside	446,0855	$C_{21}H_{19}O_{11}$
39	Kaempferol 3-O-galactoside/Quercetin 3-O-rhamnoside	447,0933	$C_{21}H_{20}O_{11}$
40	Kaempferol 3-O-glucuronide	461,0726	$C_{21}H_{18}O_{12}$
41	Kaempferol 3-O-acetyl-glucoside/Quercetin 3-O-acetyl-rhamnoside	489,1039	$C_{23}H_{22}O_{12}$

42	Kaempferol 3-O-(6''-malonyl-glucoside)/5,4'-Dihydroxy-3,3'-dimethoxy-6,7-methylenedioxyflavone 4'-O-glucuronide	533,0937	$C_{24}H_{22}O_{14}$
43	Kaempferol 3-O-xylosyl-glucoside	579,1355	$C_{26}H_{28}O_{15}$
44	Kaempferol 3-O-galactoside 7-O-rhamnoside/Kaempferol 3-O-rutinoside	593,1512	$C_{27}H_{30}O_{15}$
45	Kaempferol 3,7-O-diglucoside/Kaempferol 3-O-	609,1461	$C_{27}H_{30}O_{16}$
46	Kaempferol 3-O-(6''-acetyl-galactoside) 7-O-rhamnoside	635,1618	$C_{29}H_{32}O_{16}$
47	Kaempferol 3-O-(2''-rhamnosyl-galactoside) 7-O-	739,2091	$C_{33}H_{40}O_{19}$
48	Kaempferol 3-O-glucosyl-rhamnosyl-hexoside/Quercetin 3-O-rhamnosyl-rhamnosyl-glucoside	755,2040	$C_{33}H_{40}O_{20}$
49	Kaempferol 3-O-sophoroside 7-O-glucoside/Kaempferol 3,7,4'-O-triglucoside/Quercetin 3-O-	771,1989	$C_{33}H_{40}O_{21}$
50	Kaempferol 3-O-(2''-rhamnosyl-6''-acetyl-galactoside) 7-O-rhamnoside	783,1989	$C_{34}H_{40}O_{21}$
51	Methylgalangin	283,0612	$C_{16}H_{12}O_{5}$
52	Myricetin 3-O-arabinoside	449,0726	$C_{20}H_{18}O_{12}$
53	Myricetin 3-O-rhamnoside/Quercetin 3-O-hexoside/Quercetin 4'-O-glucoside	463,0882	$C_{21}H_{20}O_{12}$
54	Myricetin 3-O-hexoside	479,0831	$C_{21}H_{20}O_{13}$
55	Myricetin 3-O-rutinoside/Quercetin 3,4'-O-diglucoside/Quercetin 7,4'-O-diglucoside/Quercetin 3-O-sophoroside	625,1410	$C_{27}H_{30}O_{17}$
56	Phloretin	273,0769	$C_{15}H_{14}O_{5}$
57	Phloridzin	435,1297	$C_{21}H_{24}O_{10}$

58	Phloretin 2'-O-xylosyl-glucoside	567,1719	$C_{26}H_{32}O_{14}$
59	Patuletin 3-O-glucosyl-(1->6)-[apiosyl(1->2)]-glucoside	787,1939	$C_{33}H_{40}O_{22}$
60	Quercetin 3-O-glucosyl-xyloside	595,1305	$C_{26}H_{28}O_{16}$
61	Quercetin 3-O-(6"-acetyl-galactoside) 7-O-rhamnoside	651,1567	$C_{29}H_{32}O_{17}$
62	Spinacetin 3-O-glucosyl-(1->6)-glucoside	669,1672	$C_{29}H_{34}O_{18}$
63	Spinacetin 3-O-glucosyl-(1->6)-[apiosyl(1->2)]-glucoside	801,2095	$C_{34}H_{42}O_{22}$
64	Spinacetin 3-O-(2"-p-coumaroylglucosyl)(1->6)-[apiosyl(1->2)]-glucoside	947,2463	$C_{43}H_{48}O_{24}$
65	Spinacetin 3-O-(2"-feruloylglucosyl)(1->6)-[apiosyl(1->2)]-glucoside	977,2568	$C_{44}H_{50}O_{25}$
66	3,7-Dimethylquercetin	329,0667	$C_{17}H_{14}O_{7}$
67	3-Methoxysinensetin	401,1242	$C_{21}H_{22}O_{8}$
68	3-Hydroxyphloretin 2'-O-glucoside	451,1246	$C_{21}H_{24}O_{11}$
69	5,3',4'-Trihydroxy-3-methoxy-6:7-methylenedioxyflavone 4'-O-glucuronide	519,0780	$C_{23}H_{20}O_{14}$
70	3-Hydroxyphloretin 2'-O-xylosyl-glucoside	583,1668	$C_{26}H_{32}O_{15}$
Anthocyanins			
71	Cyanidin	287,0550	$C_{15}H_{11}O_{6}$
72	Cyanidin 3-O-pentoside	419,0973	$C_{20}H_{19}O_{10}$
73	Cyanidin 3-O-hexoside/Peonidin 3-O-arabinoside/Petunidin 3-O-arabinoside	449,1078	$C_{21}H_{21}O_{11}$
74	Cyanidin 3-O-(6"-succinyl-glucoside)	549,1239	$C_{25}H_{25}O_{14}$
75	Cyanidin 3-O-(6"-dioxalyl-glucoside)	592,0695	$C_{25}H_{20}O_{17}$
76	Cyanidin 3-O-(6"-p-coumaroyl-glucoside)	595,1446	$C_{30}H_{27}O_{13}$

77	Cyanidin 3-O-rutinoside	595,1658	$C_{27}H_{31}O_{15}$
78	Cyanidin 3-O-(6"-caffeoyl-glucoside)/Delphinidin 3-O-(6"-p-coumaroyl-glucoside)	611,1395	$C_{30}H_{27}O_{14}$
79	Cyanidin 3,5-O-diglucoside/Cyanidin 3-O-sophoroside/Delphinidin 3-O-rutinoside	611,1607	$C_{27}H_{31}O_{16}$
80	Cyanidin 3-O-sambubioside	616,1190	$C_{26}H_{29}ClO_{15}$
81	Cyanidin 3-O-(6"-malonyl-3"-glucosyl-glucoside)	697,1611	$C_{30}H_{33}O_{19}$
82	Delphinidin 3-O-pentoside	435,0922	$C_{20}H_{19}O_{11}$
83	Delphinidin 3-O-(6"-acetyl-hexoside)	507,1133	$C_{23}H_{23}O_{13}$
84	Delphinidin 3,5-O-diglucoside/Delphinidin 3-O-glucosyl-glucoside	627,1556	$C_{27}H_{31}O_{17}$
85	Delphinidin 3-O-6" feruloyl-glucoside	641,1501	$C_{31}H_{29}O_{15}$
86	Malvidin 3-O-glucoside	493,1341	$C_{23}H_{25}O_{12}$
87	Malvidin 3-O-(6"-acetyl-hexoside)	535,1446	$C_{25}H_{27}O_{13}$
88	Malvidin 3-O-(6"-p-coumaroyl-glucoside)	639,1708	$C_{32}H_{31}O_{14}$
89	Malvidin 3,5-O-diglucoside	655,1869	$C_{29}H_{35}O_{17}$
90	Pelargonidin	271,0601	$C_{15}H_{11}O_5$
91	Pelargonidin-3-O-[illegible]	403,1024	$C_{20}H_{19}O_9$
92	Pelargonidin 3-O-hexoside	433,1129	$C_{21}H_{21}O_{10}$
93	Peonidin 3-O-hexoside/Malvidin 3-O-arabinoside	463,1235	$C_{22}H_{23}O_{11}$
94	Pelargonidin 3-O-[illegible]	565,1552	$C_{26}H_{29}O_{14}$
95	Pelargonidin 3-O-rutinoside	579,1708	$C_{27}H_{31}O_{14}$
96	Pelargonidin 3-O-sophoroside	595,1658	$C_{27}H_{31}O_{15}$
97	Pelargonidin 3,5-O-diglucoside	630,1346	$C_{27}H_{31}ClO_{15}$
98	Peonidin	301,0707	$C_{16}H_{13}O_6$
99	Peonidin 3-O-(6"-acetyl-hexoside)	505,1341	$C_{24}H_{25}O_{12}$

100	Peonidin 3-O-(6"-p-coumaroyl-glucoside)	609,1603	$C_{31}H_{29}O_{13}$
101	Peonidin 3-O-rutinoside	609,1814	$C_{28}H_{33}O_{15}$
102	Petunidin 3-O-rhamnoside	447,1286	$C_{22}H_{23}O_{10}$
103	Petunidin 3-O-hexoside	479,1184	$C_{22}H_{23}O_{12}$
104	Petunidin 3-O-(6"-acetyl-hexoside)	521,1290	$C_{24}H_{25}O_{13}$
105	Petunidin 3-O-rutinoside	595,1658	$C_{27}H_{31}O_{15}$
106	Petunidin 3-O-(6"-p-coumaroyl-glucoside)	625,1552	$C_{31}H_{29}O_{14}$
107	Petunidin 3,5-O-diglucoside	641,1712	$C_{28}H_{33}O_{17}$

compounds in the different murta extracts.

Error (ppm)							
ASE_80-R1	ASE_80-R2	ASE_120-R1	ASE_120-R2	Conv_1-R1	Conv_1-R2	Conv_2-R1	Conv_2-R2
5,61	5,28	5,28	5,28	4,95	4,95	4,95	5,61
5,07	5,28	5,28	5,28	4,86	5,07	4,65	5,28
5,66	5,89	5,66	5,89	5,66	5,66	5,43	5,89
4,77	4,56	4,56	4,77	4,34	4,12	3,91	4,56
-5,26	-5,58	-4,95	-6,20	-5,26	-5,26	-5,26	-5,26
5,04	5,04	5,35	5,04	5,35	5,65	4,74	4,74
4,95	4,95	4,95	4,95	4,95	4,95	4,63	4,63
n.d.	n.d.	n.d.	3,19	9,75	n.d.	3,75	4,03
5,67	5,46	5,46	5,03	5,25	5,03	4,82	5,03
5,48	5,48	5,15	5,15	5,48	5,48	5,15	5,48
5,74	5,45	5,74	5,74	5,74	5,74	5,45	5,45
5,89	5,89	5,60	5,60	5,31	5,89	5,60	5,89
5,69	5,41	5,69	5,41	5,69	5,69	4,56	4,84
4,96	5,24	5,24	4,96	4,96	4,96	4,96	4,96
5,56	5,90	5,90	5,90	5,90	5,90	5,56	5,56
5,20	5,20	5,20	5,20	5,20	5,20	4,89	5,20
5,01	5,01	5,01	5,01	4,72	4,72	4,72	5,01
0,71	0,44	0,16	0,71	-0,11	0,16	0,16	0,44

5,34	5,59	5,09	5,59	5,09	5,34	5,59	5,84
7,77	7,77	9,32	9,32	0,00	-0,78	0,58	0,19
3,64	n.d.	3,78	3,20	4,36	6,95	4,07	3,92
9,49	9,76	-8,70	1,13	-3,52	-9,09	1,79	5,11
1,37	1,59	0,92	1,92	2,15	0,03	0,70	0,81
5,70	4,84	5,70	n.d.	3,12	4,30	4,52	4,84
n.d.	n.d.	n.d.	n.d.	3,09	5,27	4,13	4,13
n.d.	n.d.	n.d.	n.d.	4,80	n.d.	n.d.	n.d.
n.d.	n.d.	n.d.	n.d.	n.d.	n.d.	n.d.	n.d.
n.d.	n.d.	n.d.	n.d.	4,97	4,97	4,97	n.d.
5,05	5,05	n.d.	n.d.	5,27	5,05	4,62	n.d.
n.d.	5,02	5,02	n.d.	4,65	4,65	5,02	n.d.
4,86	n.d.	n.d.	n.d.	4,22	4,86	4,86	n.d.
4,40	4,84	4,62	4,62	4,40	4,61	4,40	4,83
4,51	4,93	4,72	4,93	4,51	4,30	4,51	4,91
4,27	4,11	n.d.	4,59	4,91	4,59	4,75	n.d.
n.d.	n.d.	n.d.	3,42	n.d.	n.d.	4,35	4,73
n.d.	n.d.	n.d.	n.d.	5,12	5,12	n.d.	n.d.
0,33	0,56	n.d.	n.d.	n.d.	n.d.	0,79	0,79
0,58	0,36	0,36	0,58	0,13	0,58	0,81	0,39
5,10	n.d.	n.d.	n.d.	5,10	5,10	5,10	n.d.
4,23	4,66	4,66	4,88	4,23	4,44	4,23	4,89
4,80	n.d.	4,80	n.d.	4,80	n.d.	n.d.	n.d.

n.d.	n.d.	n.d.	-0,98	-1,35	-0,60	-1,54	-0,23
n.d.	n.d.	4,56	5,94	3,52	n.d.	4,21	n.d.
3,69	4,20	4,54	4,70	3,69	4,20	4,87	4,37
3,46	4,45	4,28	4,45	4,12	4,12	2,64	3,30
4,50	4,66	4,66	4,03	4,50	3,89	4,98	4,50
3,92	n.d.	2,03	n.d.	4,19	1,62	3,25	2,30
4,00	4,53	3,07	n.d.	3,20	n.d.	4,13	4,13
4,84	n.d.	4,58	4,71	3,80	n.d.	4,71	4,58
n.d.	n.d.	n.d.	n.d.	3,49	n.d.	3,74	3,90
n.d.	n.d.	n.d.	n.d.	4,95	n.d.	n.d.	n.d.
n.d.	4,79	n.d.	n.d.	4,56	4,79	4,79	n.d.
n.d.	n.d.	n.d.	n.d.	4,97	n.d.	n.d.	n.d.
4,61	4,82	5,03	5,03	n.d.	n.d.	5,03	n.d.
4,51	4,19	4,51	4,51	3,87	3,71	4,03	4,67
4,58	n.d.	n.d.	n.d.	3,85	4,58	4,58	n.d.
4,99	n.d.	n.d.	n.d.	4,99	n.d.	4,99	n.d.

4,46	4,46	4,46	4,64	4,11	4,11	4,11	4,46
4,26	n.d.	4,26	4,89	4,13	4,90	4,89	4,51
4,13	4,64	4,30	4,13	3,80	3,80	3,46	4,13
2,10	n.d.	3,64	3,49	n.d.	n.d.	4,25	n.d.
4,09	n.d.	2,90	3,35	3,80	4,54	4,24	4,54
n.d.	n.d.	n.d.	n.d.	4,24	3,87	3,87	4,49
n.d.	n.d.	n.d.	n.d.	n.d.	n.d.	3,88	n.d.
-0,98	0,04	n.d.	0,14	-2,11	n.d.	-2,11	-0,67
n.d.	n.d.	n.d.	n.d.	n.d.	n.d.	4,80	n.d.
-4,26	-4,76	-4,76	-2,02	n.d.	n.d.	-4,76	-4,51
5,05	5,05	5,05	n.d.	4,83	4,83	5,05	n.d.
-1,68	-0,91	-0,91	-0,71	-0,52	-0,71	-1,48	-1,10
4,18	4,18	4,87	5,04	4,70	4,53	4,53	4,53
4,91	4,56	5,26	4,91	4,91	4,91	5,26	4,91
5,18	4,70	6,13	3,98	4,22	4,70	3,98	4,46
4,77	4,32	4,77	4,32	4,77	4,77	4,77	4,54
4,15	4,52	4,15	3,97	4,70	4,33	3,79	4,15
-2,03	-0,84	-2,70	-1,86	-1,52	-3,21	-2,03	-1,86
4,23	4,57	4,40	3,56	4,74	4,23	4,23	4,57

3,11	n.d.	n.d.	n.d.	n.d.	5,46	4,96	4,45
4,14	4,63	4,63	4,47	4,79	4,63	4,14	4,96
4,35	4,35	4,35	4,68	5,17	4,35	4,68	4,68
n.d.	n.d.	n.d.	0,41	n.d.	-0,57	-0,08	0,41
-1,08	-8,10	-7,39	-4,66	-2,80	-0,65	0,36	n.d.
4,11	4,57	4,57	4,11	4,80	4,80	4,11	4,57
4,38	4,38	3,59	4,38	4,18	4,18	4,18	3,98
4,75	4,43	4,75	4,75	4,59	5,07	4,75	4,91
4,21	4,37	4,37	4,06	4,52	4,52	4,06	4,52
4,16	4,36	4,16	4,16	4,77	4,16	4,16	4,36
3,59	3,77	3,96	3,21	3,77	4,15	3,77	4,34
n.d.	n.d.	n.d.	n.d.	4,27	4,27	4,43	4,27
-0,18	-0,79	-0,18	-0,95	1,19	0,43	0,89	0,43
4,06	4,06	4,43	4,06	4,43	4,43	4,06	4,80
4,61	4,37	3,62	4,12	4,37	4,61	4,12	4,61
3,97	4,20	3,74	4,20	4,20	4,20	4,43	4,20
4,94	4,73	4,08	4,51	4,73	4,73	4,30	4,51
4,21	6,16	4,03	2,97	3,50	4,21	3,68	3,86
4,20	4,54	4,20	4,71	4,20	4,89	3,85	4,37
n.d.	n.d.	n.d.	n.d.	4,12	3,61	4,45	4,45
-2,22	-1,59	-3,02	-0,95	-2,22	-1,43	-1,43	-1,11
5,51	5,18	4,19	5,18	4,85	4,85	4,85	4,85
-5,64	7,82	6,83	7,23	4,45	4,65	3,27	3,86

4,38	n.d.	4,22	4,22	4,22	4,05	3,89	4,55
3,78	5,42	3,61	4,76	3,78	3,94	3,61	4,43
4,41	3,51	3,96	4,41	4,18	4,85	4,41	4,41
4,17	4,17	3,97	4,17	4,38	4,17	3,76	3,97
n.d.	n.d.	5,32	2,82	5,12	n.d.	3,78	2,82
4,96	4,45	n.d.	n.d.	3,61	4,96	4,12	4,62
4,77	4,77	4,61	4,29	4,61	4,61	4,61	4,77
4,57	5,19	5,35	n.d.	4,57	5,19	4,10	5,04

DES-R1	DES-R2
5,28	5,28
5,07	5,28
5,66	5,66
4,77	4,56
-5,26	-3,71
4,74	5,65
5,59	5,27
n.d.	n.d.
5,88	5,25
5,82	5,82
6,03	5,74
5,89	5,89
5,69	5,41
5,52	5,24
5,90	6,24
5,81	5,51
5,31	5,01
n.d.	n.d.

6,85	5,34
n.d.	n.d.
n.d.	n.d.
2,72	n.d.
n.d.	n.d.
n.d.	n.d.
n.d.	n.d.
n.d.	n.d.
n.d.	5,05
n.d.	n.d.
5,05	n.d.
n.d.	4,65
n.d.	n.d.
4,84	4,84
4,51	4,30
4,43	4,75
n.d.	3,23
n.d.	n.d.
1,26	0,33
n.d.	n.d.
n.d.	n.d.
4,45	4,01
5,01	4,80

n.d.	n.d.
5,08	4,56
4,70	4,70
5,11	4,78
2,93	3,24
n.d.	3,25
4,40	n.d.
n.d.	n.d.
n.d.	n.d.
n.d.	n.d.
4,79	4,79
n.d.	n.d.
4,82	5,03
4,67	n.d.
n.d.	4,94
n.d.	n.d.

4,99	4,64
3,62	n.d.
4,64	4,47
3,79	4,71
n.d.	4,09
n.d.	n.d.
n.d.	n.d.
n.d.	n.d.
n.d.	n.d.
-4,51	-1,77
5,05	5,05
-1,10	-0,13
4,53	4,53
5,26	4,91
6,13	3,98
4,77	4,54
3,79	4,70
n.d.	n.d.
4,40	4,07

n.d.	n.d.
4,47	4,14
4,35	4,84
n.d.	n.d.
n.d.	n.d.
4,57	4,34
4,38	3,98
4,75	4,27
4,52	3,90
4,77	3,95
4,52	3,40
n.d.	n.d.
2,56	-0,64
3,69	5,16
4,61	4,12
9,74	9,28
4,73	4,73
4,21	4,39
4,71	4,37
n.d.	n.d.
n.d.	n.d.
4,85	4,85
7,82	0,89

-7,93	4,55
3,28	4,10
3,29	4,18
4,17	3,97
n.d.	n.d.
4,96	5,46
5,57	4,45
n.d.	n.d.
